# Supplementary material for: Camel Genetic Resources Conservation through Tourism: A Key Sociocultural Approach of Camelback Leisure Riding
Source: Animals (Basel). 2020 Sep 20;10(9):1703. doi: 10.3390/ani10091703 (PMC7552672; doi:10.3390/ani10091703)
Supplement: Supplementary file 1 [file animals-10-01703-s001.zip › Table S1.docx]

**Table S1:** Normality testing for customer general satisfaction and return intention probability in regards to camel tourist walks.

| **Variable** | **W'** | **V'** | **z** | **Prob>z** |
| --- | --- | --- | --- | --- |
| Customer general satisfaction | 0.989 | 1.119 | 0.232 | 0.408 |
| Return intention probability | 0.976 | 2.556 | 1.902 | 0.029 |
