# Supplementary material for: Camel Genetic Resources Conservation through Tourism: A Key Sociocultural Approach of Camelback Leisure Riding
Source: Animals (Basel). 2020 Sep 20;10(9):1703. doi: 10.3390/ani10091703 (PMC7552672; doi:10.3390/ani10091703)
Supplement: Supplementary file 1 [file animals-10-01703-s001.zip › Table S3.docx]

**Table S3.** Bayesian estimates of linear regression coefficients and model validity parameters for explanatory and predictive model comprising significantly loading factors in Dimension 3 for Customer General Satisfaction in regards to camel tourist walks.

| **Bayesian ordered logistic regression MCMC iterations** | | | 12.500 | | | |
| --- | --- | --- | --- | --- | --- | --- |
| **Random-walk Metropolis-Hastings sampling Burn-in** | | | 2.500 | | | |
| **MCMC sample size** | | | 10.000 | | | |
| **Number of obs** | | | 87 | | | |
| **Acceptance rate** | | | 0.2333 | | | |
| **Efficiency** | | | Min (0.003264) / Max (0.02403)/ Average (0.007925) | | | |
| **Log marginal-likelihood** | | | -107.36938 | | | |
| **BIC** | | | 364.090 | | | |
| **AIC** | | | -227.469 | | | |
| **AICc** | | | -227.466 | | | |
| **Customer General Satisfaction** | **Mean** | **SD** | **MCSE** | **Median** | **95% Cred. Interval** | |
| Varied | -0.0854404 | 0.1309493 | 0.015534 | -0.0885013 | -0.3341265 | 0.1817653 |
| Appropriately long | -0.2180215 | 0.1271905 | 0.008205 | -0.2216539 | -0.4632682 | 0.0322029 |
| Personal impression on involved camels’ welfare | -0.8809758 | 0.4280731 | 0.043052 | -0.8760418 | -1.712399 | -0.0418396 |
| Walk overall quality | -0.0471233 | 0.1999651 | 0.035001 | -0.0427986 | -0.4474981 | 0.3362921 |
| Familiarity towards the uses of camels in the world | 0.0407348 | 0.2085987 | 0.024233 | 0.0443482 | -0.3850831 | 0.453153 |
