# Supplementary material for: Camel Genetic Resources Conservation through Tourism: A Key Sociocultural Approach of Camelback Leisure Riding
Source: Animals (Basel). 2020 Sep 20;10(9):1703. doi: 10.3390/ani10091703 (PMC7552672; doi:10.3390/ani10091703)
Supplement: Supplementary file 1 [file animals-10-01703-s001.zip › Table S4.docx]

**Table S4.** Bayesian estimates of linear regression coefficients and model validity parameters for explanatory and predictive model comprising significantly loading factors in Dimension 6 for the Return Intention Probability in regards to camel tourist walks.

| **Bayesian ordered logistic regression MCMC iterations** | | | 12.500 | | | |
| --- | --- | --- | --- | --- | --- | --- |
| **Random-walk Metropolis-Hastings sampling Burn-in** | | | 2.500 | | | |
| **MCMC sample size** | | | 10.000 | | | |
| **Number of obs** | | | 40 | | | |
| **Acceptance rate** | | | 0.2201 | | | |
| **Efficiency** | | | Min (0.01138)/ Max (0.0229)/Average (0.01904) | | | |
| **Log marginal-likelihood** | | | -54.26764 | | | |
| **BIC** | | | 257.89 | | | |
| **AIC** | | | -233.469 | | | |
| **AICc** | | | -233.468 | | | |
| **Return Intention Probability** | **Mean** | **SD** | **MCSE** | **Median** | **95% Cred. Interval** | |
| Previous experience* | Omitted | Omitted | Omitted | Omitted | Omitted | Omitted |
| When previous experience | 0.8017737 | 0.3400471 | 0.022634 | 0.7891617 | 0.1876325 | 1.505756 |
| Comparison between experiences | 0.0039968 | 0.0492499 | 0.004617 | 0.0048556 | -0.0928177 | 0.102472 |
| *Previous experience was constant hence was omitted. | | | | | | |
